# Beyond Justice Perceptions: The Role of Interpersonal Justice Trajectories and Social Class in Perceived Legitimacy of Authority Figures

**DOI:** 10.3389/fpsyg.2021.595731

**Published:** 2021-02-12

**Authors:** Juan Liang, Xiaoyun Chen, Tian Li, Yaxin Wang

**Affiliations:** Department of Psychology, Faculty of Education, Hubei University, Wuhan, China

**Keywords:** legitimacy, interpersonal justice, trajectory, social class, fairness heuristic theory

## Abstract

There is considerable evidence that the experience of justice is associated with perceived legitimacy of authority, but there has been no research about this association when considering past rather than current fairness. Based on the fairness heuristic theory, we tested the hypothesis that interpersonal justice trajectories positively affect perceived legitimacy of the authority; we also tested whether social class moderated this effect. Community residents (*N* = 111; 54 women) rated the authority's fairness on 16 consecutive weeks and rated perceived legitimacy on the 16th week. The results of latent growth modeling showed that the trajectory of interpersonal justice scores leading up to the final week significantly predicted perceived legitimacy, regardless of the current experience of interpersonal fairness. Tests of moderation showed that the legitimacy perceptions of individuals of lower subjective social class were significantly affected by interpersonal justice trajectories, whereas this was not the case among individuals of higher subjective social class. The results are discussed in terms of their implications for research on perceived legitimacy and justice, as well as their implications for understanding social class.

## Introduction

While it is possible for authorities to shape the gains and losses of others by using power to sanction and to provide incentives, theorists, and authorities have recognized that influence over others based solely on the possession of power is costly and inefficient (Tyler, 2006). In contrast, authorities find governance will be more effective when their social influences are associated with a feeling of obligation to voluntarily obey the authorities' directives. This feeling of obligation is central to the perceived legitimacy of authority figures (Sunshine and Tyler, 2003; Jackson et al., 2012). Perceived legitimacy is the belief that the actions of an authority are appropriate, proper, and just, which is an inner motivation to accept influence (Tyler, 2006). Therefore, the perceived legitimacy provides the basis for stability and efficient management (Tyler, 2011; Jackson et al., 2012; Tyler and Jackson, 2014).

Research has consistently shown that the evaluation of the authority's fairness is a key predictor of perceived legitimacy (e.g., Van der Toorn et al., 2011; Wolfe et al., 2016). When authorities use fair procedures to make decisions (i.e., procedural justice), make fairly distributive decisions (i.e., distributive justice), or treat individuals with dignity (i.e., interpersonal justice), individuals are more likely to perceive high legitimacy (e.g., Tyler and Jackson, 2014). This empirical relationship between fairness and perceived legitimacy is evident in many contexts, such as political (e.g., Van der Toorn et al., 2011; Murphy, 2014), organizational (e.g., Blader and Tyler, 2005; Treviño et al., 2014), legal (e.g., Tyler and Jackson, 2014), and educational settings (e.g., Treviño et al., 2014; Liang and Li, 2019). Despite the wealth of research focused on issues of justice and perceived legitimacy, considerably less is understood about whether past experiences of fairness influence legitimacy perceptions. The current study addressed this issue by investigating temporal dynamics of relations between justice and perceived legitimacy over time.

Time plays an important role in the process by which justice affects perceived legitimacy. Indeed, people's evaluations of justice may change over time, showing increasing, decreasing, or static trajectories (Rubenstein et al., 2019). Recent advances suggest that justice trajectories (i.e., changes in the experience of fairness over time) exhibit a unique influence on individuals' job satisfaction and organizational commitment (e.g., Hausknecht et al., 2011; Rubenstein et al., 2019). The present study aims to extend prior research by investigating the relationship between justice trajectories and perceived legitimacy over time.

In particular, drawing on the fairness heuristic theory (Lind, 2001), we tested whether interpersonal justice trajectories positively affect perceived legitimacy of the authority. In addition, we further explored the moderating effect of social class on the effect of interpersonal justice trajectories on perceived legitimacy. Specifically, we expected that the effect of interpersonal justice trajectories on perceived legitimacy will be stronger for lower-class individuals. In what follows, we first elaborate the role of interpersonal justice trajectories in the formation of legitimacy perceptions. We then address the presumed moderating impact of social class.

In the current study, we focused on interpersonal justice, which refers to perceptions about the extent to which the authorities treat people with sensitivity, dignity, and respect (Bies, 2005). Compared to the fairness of decision outcomes (i.e., distributive justice) and decision-making processes (i.e., procedural justice), interpersonal justice can occur in daily interactions between individuals and authorities (Bies, 2005). For example, the authorities' words, expressions, and attitudes may directly communicate interpersonal justice information. These daily interactions are important for the construction of perceived legitimacy (Tyler, 2011). Moreover, of these three forms of justice, interpersonal justice is likely to fluctuate the most over time (Johnson et al., 2014; Scott et al., 2014), because authorities tend to have more discretion in whether to adhere to interpersonal justice rules than distributive and procedural rules (Scott et al., 2009).

### Interpersonal Justice Trajectories and Legitimacy Perceptions

The traditional explanation of the relationship between justice and perceived legitimacy is that when a person believes that the authority is treating them fairly, they may also believe that they have a high-quality relationship with the authority and a good status within the group (Tyler, 2006). This relational information thus represents a kind of exchange resource that the authority provides in interactions with the person (Tyler, 1997; Colquitt et al., 2012; Liang and Li, 2019). In order to ensure and strengthen the relationship (Chen et al., 2014), the individual may reciprocate by legitimating the authority (Tyler, 1996) and thus perceive high legitimacy (Tyler, 1996; Liang and Li, 2019). The high legitimacy perceptions in turn engender a desire to voluntarily comply and cooperate with the authority and to engage within the group of governed persons (Tyler and Jackson, 2014). However, it should be noted that such exchange relationships mature over time (Blau, 1964; Colquitt et al., 2013). Legitimacy perceptions are the result of fairness the person experiences in a series of person–authority social interactions (Tost, 2011; Tyler, 2011).

Drawing on the fairness heuristic theory (Lind, 2001), we propose that interpersonal justice trajectories may provide independent information useful for predicting future perceived legitimacy. The fairness heuristic theory posits that people not only would like to commit themselves to authorities but are also worried about being exploited (Lind, 2001). As such, people will engage in a “judgment” phase in which they construct fairness heuristics or cognitive shortcuts when deciding whether to trust an authority. Individuals then move quickly into the “use” phase in which the heuristic is used to determine whether to commit to and cooperate with an authority (Lind, 2001). Fairness heuristics anchor the interpretations of subsequent information, with these interpretations being typically biased in the direction of the initial heuristic (Lind, 2001). On the basis of the fairness heuristic theory, individuals would be expected to construct a heuristic about an authority once they have formed interpersonal justice trajectories (Hausknecht et al., 2011). That is, people may utilize their interpersonal justice trajectories as a heuristic when they make sense of the authority's legitimacy.

Based on the fairness heuristic theory, we assume that if an individual experiences a historical upward interpersonal justice trajectory, they will perceive the authority as more legitimate. That is, the individual interprets the upward trend as a signal of improvements in the authority's contributions to the exchange relationship, such as greater respect and care (Rubenstein et al., 2019), which in turn might strengthen the reciprocal interpersonal relations (Colquitt et al., 2012; Chen et al., 2014). The individual's reciprocation then is marked by legitimating the authority (Tyler, 1996) and thus high perceived legitimacy (Tyler, 1996; Liang and Li, 2019).

Conversely, if evaluations of interpersonal justice trend downward, we assume that the individual will commensurately be less inclined to perceive legitimacy. Declines in the authority's respect and genuine care suggest to the person that the interpersonal relationship is becoming progressively bleaker (Lindsley et al., 1995; Ariely and Carmon, 2000); as a result, the person may become less invested in the social exchange relationship and will withhold compliance to the authority (Blau, 1964; Rubenstein et al., 2019).

Hypothesis 1—interpersonal justice trajectories positively affect perceived legitimacy of the authority.

### Moderating Role of Social Class

Given the centrality of legitimacy in many real-world situations, it is important to understand the conditions under which interpersonal justice trajectories are more likely or less likely to influence perceived legitimacy. A limitation of many studies in this literature is a lack of attention to the specific conditions under which justice trajectories may be more or less important for individuals (e.g., Hausknecht et al., 2011; Rubenstein et al., 2019). In other words, these studies assume that justice trajectories are equally salient to most individuals, which may or may not be the case. We argue that social class may moderate the effect of interpersonal justice trajectories on perceived legitimacy of the authority. Social class is a core aspect of how people think of “self” and relate to the social world (Kraus et al., 2012). With the dramatic social development, the divisions between social classes are becoming wider (Yang et al., 2017). Social class may shape individuals' social attitudes and behaviors (Brown-Iannuzzi et al., 2015). For example, Brandt (2013) found that, relative to their higher social class counterparts, lower social class individuals had lower trust and confidence in government and social institutions. Furthermore, individuals from different social classes may employ different social cognitive patterns to construct their social environments (Kraus et al., 2012). Hence, understanding how lower and higher social class individuals construct authorities' legitimacy has important implications for social management.

It should be noted that social class comprises both objective characteristics and subjective perceptions (Kraus et al., 2009). The objective characteristics of social class are measured by indicators of material wealth, including a person's educational attainment, income, or occupational prestige (Kraus et al., 2009, 2012). Subjective perceptions of social class capture the psychological experience of rank underlying a person's social class (Kraus et al., 2009). Some have proposed that subjective social class may be a more potent predictor of social cognitive tendencies and physiological health outcomes (Kraus et al., 2009). Hence, in the present investigation, we study the moderating impact of subjective social class in the relationships between interpersonal justice trajectories and perceived legitimacy.

Social class has been said to moderate the effect of interpersonal justice trajectories on perceived legitimacy for two reasons. First, individuals of a lower social class may be more sensitive to variations in justice compared to individuals of a higher social class. The life circumstances of individuals of a lower social class, such as poor socioeconomic conditions, induce significant uncertainties (Kraus et al., 2012; Diehl et al., 2016). According to the uncertainty management theory and related empirical studies (Van den Bos and Lind, 2002; Colquitt and Zipay, 2015), individuals of a lower social class may reduce this uncertainty by focusing more on fairness information than individuals of a higher social class do. Thus, individuals of a lower social class may be more sensitive to issues of justice compared with individuals of a higher social class.

Second, lower social class individuals are more likely to use interpersonal justice trajectories as a kind of information to form legitimacy perceptions. Although justice trajectories can provide relational information (such as information about relationship quality with the authority) (Tyler and Lind, 1992), this type of information may be important only to the extent that people define the “self” through their social relationships (De Cremer et al., 2008). Individuals of a lower social class tend to develop communal self-concepts (Kraus et al., 2012) and to conceive of the self as defined by social connections to other individuals, important social groups, and communities (Kraus et al., 2012). Communal self-concepts therefore should make lower social class individuals more likely to use the experience of justice as a reflection of the self, which may engender more sensitive reactions to interpersonal justice trajectories.

In contrast, individuals of a higher social class are more likely to develop personally agentic self-concepts and to conceptualize the self in terms of individual agency, personal choice, autonomy, and uniqueness relative to others (Kraus et al., 2012). In this group, justice trajectories should be less important to building a positive sense of self, in which case a weaker reaction to interpersonal justice trajectories should be expected.

In addition, from a cognitive perspective, lower social class individuals are more likely to use interpersonal justice trajectories as a kind of social heuristic to form legitimacy perceptions. Lower social class individuals may pay greater attention to the entire perceptual field, especially relations among objects and events, and a tendency to give contextual explanations for social events (Nisbett et al., 2001; Kraus et al., 2009). Therefore, individuals of the lower social class may be more likely to use their interpersonal justice trajectories, as a kind of social context, to make judgments. In contrast, higher social class individuals are more likely to ascribe causality to focal actors or objects and less likely to give contextual explanations for social events (Grossmann and Varnum, 2011). Hence, the higher social class may show relatively low sensitivity to interpersonal justice trajectories in relation to perceived legitimacy of the authority.

Hypothesis 2—Social class moderates the effect of interpersonal justice trajectories on legitimacy perceptions; individuals of a lower social class will be more strongly affected by interpersonal justice trajectories than individuals of a higher social class.

## Methods

We used a longitudinal design to test the hypotheses. To track participants' interpersonal justice trajectories, we surveyed a sample of residents about their justice experiences at four time points over 16 weeks. The time between the follow-ups was 4 weeks. We assessed the participants' self-rated social class at Time 1, and we assessed their perceptions of the authority's legitimacy at Time 4.

### Participants and Procedure

The residents were volunteers who were asked to complete questionnaires. We used a snowball sampling procedure that resulted in a total of 140 residents who completed the questionnaires. All participants provided written informed consent. The study was approved by the institutional review board (ethics committee) at the university with which the authors are affiliated. Of all the participants at Time 1, 89.29, 85, and 79.29% completed questionnaires at Times 2, 3, and 4, respectively. Thus, the final sample consisted of 111 participants with valid data (54 female, *M*_age_ = 39.45, SD = 11.96). Table 1 displays demographic information.

**Table 1:** Sociodemographic data of sample.

**Variable**	**% (*N*)**
Education level (*N* = 111)	
Primary school and below	15 (13.5)
Junior high school	31 (27.9)
Senior high school	12 (10.8)
Junior college	9 (8.1)
College	29 (26.1)
More than college	14 (12.6)
Not reported	1 (0.9)
Occupational status (*N* = 111)	
State & society managerial	17 (15.3)
Managerial	5 (4.5)
Corporate	8 (7.2)
Professional	26 (23.4)
Clerical	5 (4.5)
Enterprise	7 (6.3)
Service	9 (8.1)
Worker	9 (8.1)
Farming	18 (16.2)
Unemployed	7 (6.3)

We conducted two G-power analyses (i.e., sensitivity test). The configuration parameters in G^*^Power version 3.1 (Faul et al., 2007) were as follows. For data that was used in latent growth modeling (LGM), the power was set at 0.99, the alpha level of two-tailed tests was set at 0.05, the total sample size was 111, the number of groups was set at 1, and the number of measurements was set at 4. Based on these parameters, the effect size was 0.16. For data that was used in moderating regression analysis, the power was set at 0.99, the alpha level of two-tailed tests was set at 0.05, the total sample size was 111, and the number of predictors was set at 4. The result showed that the effect size was 0.24. Hence, the G^*^power analyses indicated that the sample size was sufficient to detect a small to medium effect size.

### Measures

#### Interpersonal Justice

We assessed interpersonal justice with three items developed by Colquitt (2001), at each of the four time points: “The local government officials treated me with patience,” “The local government officials treated me with dignity,” and “The local government officials treated me with respect” (1 = *strongly disagree* to 5 = *strongly agree*). Cronbach alpha coefficients for the items were 0.90, 0.90, 0.86, and 0.91 at Time 1, Time 2, Time 3, and Time 4, respectively.

#### Subjective Social Class

We used the Subjective Social Status Scale to assess the participants' self-reported social class (Adler et al., 2000). Participants were shown a drawing of a ladder with 10 rungs (Kraus et al., 2010) and were told, “Think of the ladder above as representing where people stand in the important groups to which they belong. The higher rung means the higher social class.” Then they were asked: “Where do you stand in relation to others on income, education and occupation?” (1 = *bottom rung* to 10 = *top rung*). Subjective social class measured at Time 1 was the moderator variable in the analyses.

#### Objective Social Class

As in previous research, we operationalized objective social class as the participants' educational attainment (Kraus et al., 2009) and occupation status (Adler et al., 2000). Educational attainment was assessed using six categories: (1) primary school and below, (2) junior high school, (3) senior high school, (4) junior college, (5) college, and (6) more than college. Higher numbers indicated greater educational attainment. Occupation status was assessed using 10 categories: (1) state and society managerial, (2) managerial, (3) corporate, (4) professional, (5) clerical, (6) enterprise, (7) service, (8) worker, (9) farming, and (10) unemployed. Higher numbers indicated lower occupation status. Distributions of these variables are shown in Table 1.

#### Perceptions of the Authorities' Legitimacy

Perceived legitimacy was measured using two items: “I would unquestioningly accept the local government's decisions,” and “I should voluntarily comply with the local government's decisions” (1 = *strongly disagree* to 5 = *strongly agree*) (Van der Toorn et al., 2011) (*r*_s_ = 0.48, *p* < 0.01).

### Statistical Analysis

The statistical analyses consisted of two stages. In the first stage, we employed latent growth modeling (LGM; Chan, 1998; Chan and Schmitt, 2000) to assess the changes in interpersonal justice perceptions over time (Mplus Version 7.2; Muthén and Muthén, 2012). With LGM, items at each time point are used to create a distinct latent intercept (i.e., initial status) and trajectory (i.e., slope or change over time).

An important assumption underlying LGM is that one is measuring the same substantive constructs over time—termed measurement invariance. Following procedures outlined by Chan (1998), before conducting the LGM, we first compared the fit statistics of two models. The first model freely estimated factor loadings for each variable at each time point (i.e., not specified to be any value, other than the initial factor set at 1). The model fit was as follows: χ^2^ = 105.78, df = 48; CFI = 0.95, TLI = 0.92, and RMSEA = 0.1. In the second model, the estimates were invariant, or constrained, meaning that factor loadings for each indicator were fixed to be equal. The model fit was as follows: χ^2^ = 113.15, df = 56; CFI = 0.95, TLI = 0.94, and RMSEA = 0.1. A similar fit between the two models indicates support for invariance (Chan, 1998). Thus, we can be confident in measurement invariance over time. We then used Mplus to save each participant's interpersonal justice trajectory factor scores (using the SAVE = FSCORES command). This command returns a value for each participant representing the amount of interpersonal justice change over the course of the survey period.

In the second stage of analyses, we tested the moderating effect of subjective social class in the relationship between interpersonal justice trajectories and perceived legitimacy of authority. For this analysis, we used Model 1 in the PROCESS macro for SPSS (Hayes, 2013) with 5,000 bootstrapped samples.

The second stage of statistical analyses can be summarized as follows. The interpersonal justice trajectory was the independent variable, and perceived legitimacy was the dependent variable. Subjective social class was the moderator variable. The justice evaluation at Time 4 and age were included as the covariates (Brienza and Ramona, 2017; Rubenstein et al., 2019).

## Results

### Descriptive Statistics

The means, standard deviations, and correlations among all variables are shown in Table 2.

**Table 2:** Means, standard deviations, and correlation matrix of observed variables.

	***M***	**SD**	**1**	**2**	**3**	**4**	**5**
1. Subjective social class (Time 1)	4.48	1.75					
2. Interpersonal justice (Time 1)	3.92	0.89	0.08				
3. Interpersonal justice (Time 2)	3.84	0.88	0.10	0.65^**^			
4. Interpersonal justice (Time 3)	3.73	0.95	0.24^*^	0.46^**^	0.53^**^		
5. Interpersonal justice (Time 4)	3.69	0.98	0.24^*^	0.44^**^	0.47^**^	0.71^**^	
6. Perceptions of legitimacy (Time 4)	3.36	1.01	0.10	0.05	0.07	0.32^*^	0.43^**^

*N = 111*.

* p < 0.05,

** *p < 0.01, two-tailed tests*.

### Latent Growth Model

Before testing the hypotheses, we first examined parameter estimates of the growth model to test whether participants' interpersonal justice scores changed significantly over the course of the survey period. These results are presented in Table 3. The results showed that the *mean* trajectories for interpersonal justice were significant, indicating significant change in mean scores from Time 1–4 across all participants. Importantly, the between-person interpersonal justice trajectory *variance* was significant, meaning that some participants' scores showed a significantly improving interpersonal justice trajectory, some showed a worsening interpersonal justice trajectory, and others showed a stagnant interpersonal justice trajectory (Rubenstein et al., 2019). Overall, this significant variance component shows that justice evaluations did significantly change over time, thereby allowing for this variable to be tested as an independent variable.

**Table 3:** Significance tests for latent growth model parameters.

	**Mean level**	**Variance in level**	**Mean slope**	**Variance in slope**
Interpersonal justice	3.91^***^	0.57^***^	−0.08^**^	0.07^***^

*N = 111. Level represents the average rating of interpersonal justice across Times 1 through 4*.

** p < 0.01,

*** *p < 0.001, two-tailed tests*.

### Perceptions of Legitimacy

All results are shown in Table 4. Hypothesis 1 proposed that interpersonal justice trajectories would predict perceptions of authorities' legitimacy. After controlling for interpersonal justice perceptions at Time 4 and age, increases over time in interpersonal justice were related to significantly higher levels of perceptions of legitimacy (*b* = 1.25, SE = 0.52, *t* = 2.01, *p* < 0.05). Thus, Hypothesis 1 was supported.

**Table 4:** Results of interpersonal justice trajectory, subjective social class, and interactions predicting perceptions of legitimacy.

**Predictor**	***b***	**SE**	***t***	***p***	**95% CI**
Constant	1.68	0.49	3.41	0.001	[0.71, 2.67]
Interpersonal justice (Time 4)	0.28	0.11	2.43	0.02	[0.05, 0.51]
Age	0.18	0.01	2.43	0.02	[0.003, 0.03]
Interpersonal justice trajectory	1.25	0.52	2.41	0.02	[0.22, 2.28]
Subjective social class	0.01	0.05	0.26	0.80	[−0.09, 0.11]
Interpersonal justice trajectory × subjective social class	−0.54	0.21	−2.51	0.01	[−0.96, −0.11]
Model *R*^2^ *=* 0.29 (*p* < 0.001)					
Conditional effect of the interpersonal justice slope at value of moderator (subjective social class)					
−1 SD (−1.75)	2.19	0.63	3.50	0.007	[0.95, 3.43]
*M* (0.00)	1.25	0.52	2.41	0.02	[0.22, 2.28]
1 SD (1.75)	0.31	0.65	0.47	0.64	[−0.99, 1.60]

*N = 111. Bootstrap sample size = 5,000. CI, confidence interval*.

**Figure 1:**
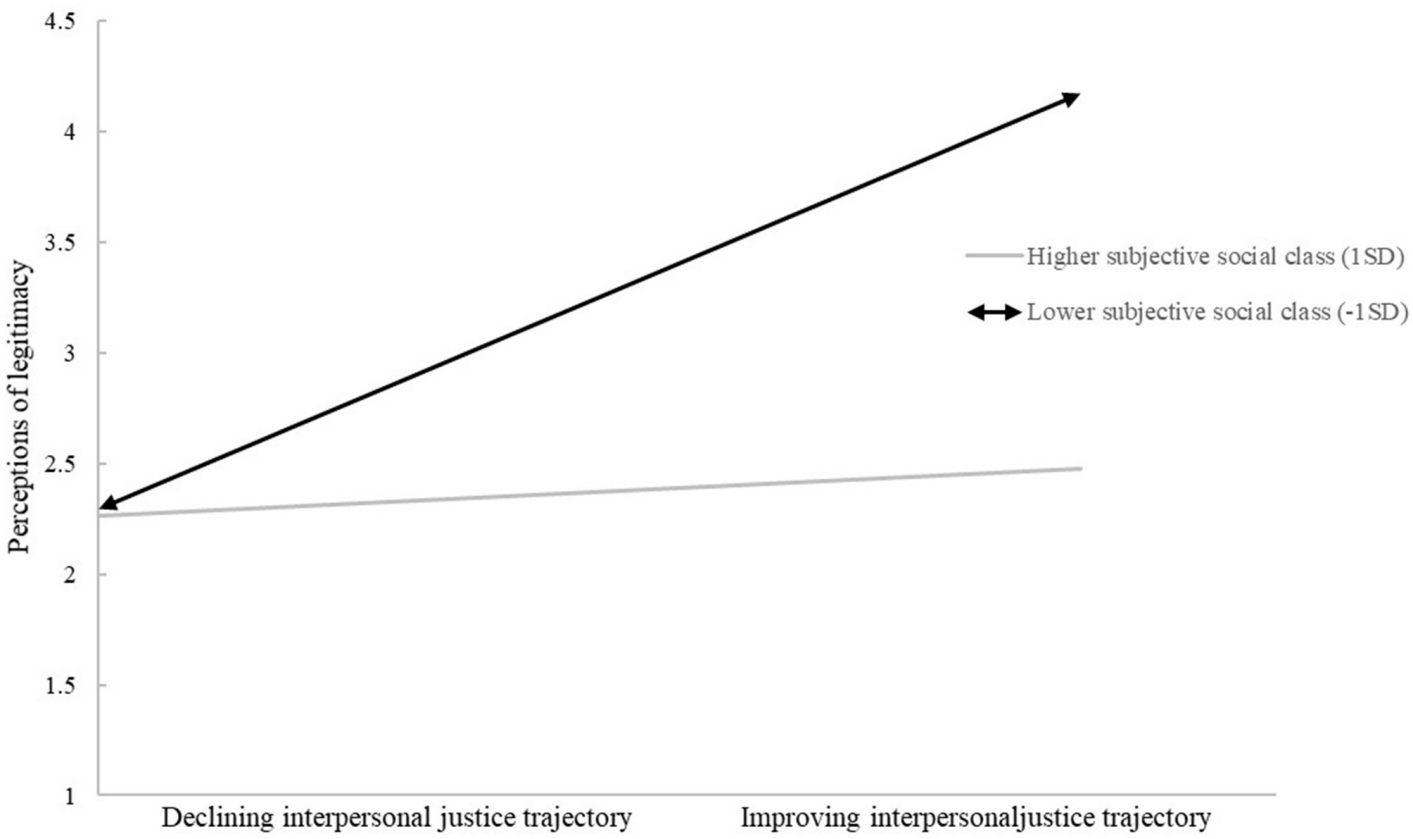
Perceptions of legitimacy as a function of interpersonal justice trajectory and subjective social class. Means are on 5-point scales with higher scores indicating higher perceived legitimacy.

The results also showed a significant interaction between interpersonal justice trajectory and subjective social class in predicting perceptions of legitimacy (*b* = −0.54, SE = 0.21, *t* = −2.51, *p* < 0.05). *Post-hoc* analyses further showed that for residents of lower subjective social class (1 SD below the mean), interpersonal justice trajectory had a significant effect on perceived legitimacy [conditional effect = 2.19, SE = 0.63, 95% CI bias corrected (0.95, 3.43)], whereas for residents of higher subjective social class (1 SD above the mean), the effect of interpersonal justice trajectory on perceptions of legitimacy was not significant [conditional effect = 0.31, SE = 0.65, 95% CI bias corrected (−0.99, 1.60)]. Consistent with our prediction, the legitimacy perceptions of individuals of the lower subjective social class were significantly affected by interpersonal justice trajectory at *p* < 0.01, whereas this was not the case among individuals of higher subjective social class (see Figure 1). Hence, Hypothesis 2 was also supported.

Given that social class includes both subjective and objective facets, it is necessary to analyze the moderating effects of objective social class in the relationships between interpersonal justice trajectories and perceived legitimacy. We conducted two moderation analyses. The interpersonal justice trajectory was the independent variable, perceived legitimacy was the dependent variable, and educational attainment or occupation status was the moderator variable. The interpersonal justice perceptions at Time 4 and age were control variables. We reported these results in Table 5. When educational attainment was included as a moderator, the main effect of interpersonal justice trajectory was not significant (*b* = 0.83, SE = 0.52, *t* = 1.59, *p* = 0.11), but the interaction effect was significant (*b* = −0.57, SE = 0.23, *t* = −2.51, *p* < 0.05). *Post-hoc* analyses further showed that for residents of lower educational attainment (1 SD below the mean), interpersonal justice trajectory had a significant effect on perceived legitimacy [conditional effect = 1.80, SE = 0.57, 95% CI bias corrected (0.68, 2.93)], whereas for residents of higher educational attainment (1 SD above the mean), the effect of interpersonal justice trajectory on perceptions of legitimacy was not significant [conditional effect = −0.14, SE = 0.72, 95% CI bias corrected (−1.57, 1.29)].

**Table 5:** Results of interpersonal justice trajectory, objective social class, and interactions predicting perceptions of legitimacy.

**Predictor**	***b***	**SE**	***t***	***p***	**95% CI**
Educational attainment (*N* = 110)					
Constant	1.68	0.48	3.49	0.001	[0.72, 2.63]
Interpersonal justice (Time 4)	0.34	0.11	3.08	0.002	[0.12, 0.56]
Age	0.01	0.01	1.58	0.11	[−0.002, 0.03]
Interpersonal justice trajectory	0.83	0.52	1.59	0.11	[−0.20, 1.86]
Educational attainment	−0.13	0.05	−2.58	0.01	[−0.22, −0.03]
Interpersonal justice trajectory × educational attainment	−0.57	0.23	−2.51	0.01	[−1.01, −0.12]
Model *R*^2^ *=* 0.34 (*p* < 0.001)					
Conditional effect of the interpersonal justice slope at value of moderator (educational attainment)					
−1 SD (−1.72)	1.80	0.57	3.17	0.002	[0.68, 2.93]
*M* (0.00)	0.83	0.52	1.59	0.11	[−0.20, 1.86]
1 SD (1.72)	−0.14	0.72	−0.20	0.84	[−1.58, 1.29]
Occupation status (*N* = 111)					
Constant	1.61	0.50	3.19	0.002	[0.61, 2.60]
Interpersonal justice (Time 4)	0.32	0.12	2.79	0.006	[0.09, 0.55]
Age	0.14	0.01	2.00	0.05	[0.001, 0.03]
Interpersonal justice trajectory	1.07	0.54	2.00	0.05	[0.01, 2.14]
Occupation status	0.06	0.03	2.27	0.03	[0.01, 0.12]
Interpersonal justice trajectory × occupation status	0.12	0.15	1.04	0.30	[−0.14, 0.44]
Model *R*^2^ *=* 0.29 (*p* < 0.001)					

*Bootstrap sample size = 5,000. CI, confidence interval*.

When occupation status was modeled as a moderator, there was a significant main effect of interpersonal justice trajectory (*b* = 1.07, SE = 0.54, *t* = 2.00, *p* = 0.05), but no significant interaction between interpersonal justice trajectory and occupation status (*b* = 0.15, SE = 0.15, *t* = 1.04, *p* = 0.30).

## General Discussion

Research to date has shown a concurrent relationship between the experience of justice and perceptions of an authority's legitimacy. This study examined this relationship when prior experiences with justice are taken into account. The trajectory of interpersonal justice over the course of 16 weeks predicted perceived legitimacy of the authority at week 16. This association was significant for individuals who rated themselves as being lower vs. higher in subjective social class. These results have both theoretical and practical implications.

### Implications

First, we contribute to the justice–legitimacy literature by taking a temporal dynamic perspective and focusing on the effect of interpersonal justice trajectories on perceived legitimacy. Whereas, previous research focused on the effect of individuals' justice perceptions on their legitimacy perceptions by measuring or manipulating justice at one time point (e.g., Tyler and Jackson, 2014; Tankebe et al., 2016), the current study utilized the temporal dynamic approach to examine the role of justice trends in the legitimacy construction process over time. The present findings contribute to theory on legitimacy by demonstrating that individuals' legitimacy perceptions of authority do not occur in a vacuum, but rather are shaped through time.

Second, the results of our study identified a boundary condition (i.e., subjective social class) of the justice trajectory effect. To our knowledge, this is the first study on this question. Previous studies found that justice trajectories exhibited a unique influence in predicting job satisfaction, organizational commitment, helping behavior, and turnover (Hausknecht et al., 2011; Rubenstein et al., 2019). Our research furthers these studies and highlights the important role of people's subjective social class in accounting for whether people use interpersonal justice trajectories to form their legitimacy perceptions. Moreover, it would be useful to develop temporal dynamic justice models that include information about both justice (e.g., current justice levels and trajectories) and individual characteristics (e.g., social class). This would result in a more complete account of the mechanisms underlying the justice trajectory effect.

Third, the current study also advances the research on social class and justice area by considering social class as a moderator. While previous research has shown that social class influences diverse domains that include subjective well-being (e.g., Yu and Blader, 2019), prosocial behavior (e.g., Kraus et al., 2017), unethical behavior (e.g., Dubois et al., 2015), and cognitive performance (e.g., Nisbett et al., 2001), our findings suggest that subjective social class may play a significant role in the formation of legitimacy perceptions and in the justice trajectory effect—topics that have received little attention to date. These findings suggest that social class shapes the psychological process underlying the formation of judgments about authority, thus deepening our understanding of the psychology of social class. Finally, the significant effect of lower subjective social class on the positive relationship between interpersonal justice trajectories and legitimacy perceptions is consistent with the idea that people rely on fairness heuristics to make judgments only when they are sensitive to these heuristics (Van den Bos and Lind, 2002; Greifeneder et al., 2011).

It is worth noting that the association between interpersonal justice trajectories and perceived legitimacy did not emerge as significant among higher subjective social class participants. This may be because of the higher social class individuals' social cognitive patterns. Higher social class individuals enjoy a sense of certainty (Diehl et al., 2016) and perceive themselves to hold high status and power (Yu and Blader, 2019), which may lead them to be insensitive to justice information and unlikely to use social heuristics when making judgments (Van den Bos and Lind, 2002; De Cremer et al., 2008; Greifeneder et al., 2011). Hence, they may be less likely to rely on interpersonal justice trajectories to form their perceptions of legitimacy. Future research could examine this assumption. Another interesting question for future research is which source of information higher social class individuals will draw on when forming legitimacy perceptions.

The results of our study also have practical implications. Given the large number of studies on the justice–legitimacy relationship, it is surprising how little research has focused on individual differences (such as social class). Some studies have indicated that lower social class individuals tend to show distrust in many forms of social interaction with authorities and widespread disengagement from political systems (Kraus et al., 2012, 2017). The present findings suggest that individuals in the lower subjective social class may be more negatively influenced by a declining interpersonal justice trajectory than individuals in the higher subjective social class. Government authorities may need to work harder to establish and maintain relationships with members of the lower subjective social class, who may otherwise be reluctant to invest in government initiatives.

### Limitations and Future Directions

The limitations of this study should be acknowledged. The first potential limitation is that we did not assess the underlying reason that social class would moderate the association between interpersonal justice trajectories and legitimacy perceptions. For instance, we assumed that lower social class individuals are more sensitive to fairness variations because noticing these changes can reduce uncertainty. Moreover, we assumed that lower social class individuals are more likely to rely on interpersonal justice trajectory information to construct their sense of self. It would be desirable to have further evidence that the results emerged for the reasons we assumed.

The second potential limitation concerns the methodology we employed. Although a key strength was the longitudinal design, all data were based on self-reports. This may raise the concern that the correlations were artificially inflated by common method variance, a possibility that was mitigated to some degree because there was temporal separation of measurement (Podsakoff et al., 2003). It also should be noted that, although the sample size had acceptable statistical power, it was nevertheless relatively small. We thus encourage future researchers to explore the role of social class in the relationship between interpersonal justice trajectories and perceived legitimacy of authority with a bigger sample size.

Third, we did not address the question of whether current evaluations of interpersonal justice are more or less important than justice trajectories in shaping perceived legitimacy. This question is especially interesting because the relative importance of these two variables may be moderated by both the social context and individual differences.

While we mainly focused on of the individual difference variable of subjective social class as a moderator of the effect of interpersonal justice trajectories, future research could also examine the roles of contextual moderators. For macro-level social context variables, it would be useful for future researchers to examine whether and how group justice climate (Naumann and Bennett, 2000) and cultural differences (Blader, and Tyler, 2005) influence the interpersonal justice trajectory effect. For micro-level context variables, future researchers can explore leadership (e.g., ethical leadership) (Koopman et al., 2019) and the individual–authority relationship (e.g., trust) (Colquitt et al., 2012) as moderators.

## Conclusion

Despite the considerable evidence documenting the relationship between fairness and perceived legitimacy, considerably less is understood about how legitimacy perceptions are influenced by individuals' past experiences of fairness. We demonstrated that past experiences of interpersonal fairness—namely, interpersonal justice trajectories—have an important impact on legitimacy perceptions. Furthermore, the positive relationship between interpersonal justice trajectories and legitimacy perceptions was significant for individuals of lower vs. higher subjective social class individuals.

## Data Availability Statement

The raw data supporting the conclusions of this article will be made available by the authors, without undue reservation.

## Ethics Statement

The studies involving human participants were reviewed and approved by all observers provided informed written consent. The study was approved by the institutional review board (ethics committee) of the Faculty of Education at Hubei University. The patients/participants provided their written informed consent to participate in this study.

## Author Contributions

JL conceived and designed the research and analyzed the data. JL, XC, TL, and YW performed the longitudinal survey. JL, XC, and TL contributed to the writing of the manuscript. All authors contributed to the article and approved the submitted version.

## Conflict of Interest

The authors declare that the research was conducted in the absence of any commercial or financial relationships that could be construed as a potential conflict of interest.
